# Red Cell Distribution Width as a Predictor of Survival in Patients with Hepatocellular Carcinoma

**DOI:** 10.3390/medicina60030391

**Published:** 2024-02-25

**Authors:** Gianpaolo Vidili, Angelo Zinellu, Arduino Aleksander Mangoni, Marco Arru, Valentina De Murtas, Elena Cuccuru, Alessandro Fancellu, Panagiotis Paliogiannis

**Affiliations:** 1 Department of Medicine, Surgery and Pharmacy, University of Sassari, Viale San Pietro 43a, 07100 Sassari, Italy; gvidili@uniss.it (G.V.); m.arru33@phd.uniss.it (M.A.); vale.demu@yahoo.it (V.D.M.); elena.cuccuru@aouss.it (E.C.); afancel@uniss.it (A.F.); 2 Department of Biomedical Sciences, University of Sassari, Viale San Pietro 43b, 07100 Sassari, Italy; azinellu@uniss.it; 3 Discipline of Clinical Pharmacology, College of Medicine and Public Health, Flinders University, Bedford Park, SA 5042, Australia; arduino.mangoni@flinders.edu.au; 4 Department of Clinical Pharmacology, Flinders Medical Centre, Southern Adelaide Local Health Network, Bedford Park, SA 5042, Australia

**Keywords:** liver, cancer, hepatocellular carcinoma, HCC, biomarkers, RDW, blood

## Abstract

*Background and Objectives.* Hepatocellular carcinoma (HCC) and the intrahepatic biliary tract cancers are estimated to rank sixth for incidence among solid cancers worldwide, and third for mortality rates. A critical issue remains the need for accurate biomarkers for risk stratification and overall prognosis. The aim of this study was to investigate the ability of a biomarker of heterogeneity of the size of red blood cells, the red cell distribution width (RDW), to predict survival in patients with HCC. *Materials and Methods.* A consecutive series of patients with a histologic diagnosis of HCC were included into this study irrespective of their age, stage of the disease, and treatment administered, and followed-up for a period of three years. Demographic, anthropometric [age, sex, body mass index (BMI)], and clinical data (Charlson Comorbidity Index, Child–Pugh score, etc.), along with laboratory tests were retrieved from clinical records. *Results.* One-hundred and four patients were included in this study. Among them, 54 (69%) were deceased at the end of the follow-up. Higher RDW values, but not other hematological and biochemical parameters, were significantly associated with mortality in both univariate and multivariate analysis. The optimal RDW cut-off value identified with the Youden test for survival was 14.7%, with 65% sensitivity and 74% specificity (AUC  =  0.718, 95% CI 0.622–0.802, *p*  <  0.001). Kaplan–Meier survival curves showed significantly lower survival with higher RDW values (HR = 3.5204; 95% CI 1.9680–6.2975, *p* < 0.0001) with a mean survival of 30.9 ± 9.67 months for patients with RDW ≤ 14.7% and 22.3 ± 11.4 months for patients with RDW > 14.7%. *Conclusions.* The results of our study showed that RDW can perform better than other blood-based biomarkers in independently predicting prognosis in patients with HCC.

## 1. Introduction

Hepatocellular carcinoma (HCC), together with the intrahepatic biliary tract cancers, is estimated to rank sixth for incidence among solid cancers worldwide, and third for mortality rates, with more than 900,000 new cases and more than 830,000 deaths in 2020, according to the Global Cancer Observatory (GLOBOCAN) data [1]. Albeit the prevalence of HCC is highest in Eastern Asia and sub-Saharan Africa, where chronic hepatitis B infection is endemic and the food is often contaminated with the mycotoxin aflatoxin B1, at least one quarter of the cases is idiopathic; recent cancer epidemiology reports have shown an increasing incidence of liver cancer in Western countries in the recent two decades, especially in women [2,3]. The close link between incidence and mortality rates reflects the lack of effective screening strategies, difficulties in early diagnosis, and poor outcomes with current therapeutic options, especially when diagnosed in advanced stage and in patients with compromised liver function and general clinical conditions. Patients with advanced stage disease are generally treated with multidisciplinary therapies, including surgical, locoregional, systemic, and supportive treatment options encompassing several specialties comprising surgery, radiology, oncology, hepatology, and palliative care. A critical issue, however, remains the need for accurate risk stratification in order to select the best treatment option and predict clinical outcomes. Circulating biomarkers such as bilirubin and albumin are useful for the classification of the underlying cirrhosis, and alpha fetoprotein is used in combination with other clinical and imaging factors for disease stage, while numerous other biomarkers are currently under evaluation [4].

Complete blood count-based biomarkers have been shown to correlate well with clinical outcomes in numerous chronic [5,6,7,8,9,10], infectious [11,12,13,14,15], and surgical conditions [16,17,18,19,20], as well as in cancer [21,22,23,24,25,26]. In particular, the potential role of the red cell distribution width (RDW), a biomarker of the heterogeneity of red blood cells’ size, in predicting adverse events and survival currently represents a topic of intense research in numerous clinical conditions [27,28], including malignancies such as colorectal cancer [29,30,31], esophageal cancer [32,33], lung cancer [34,35], breast cancer [36,37], and other malignancies [38,39,40,41,42]. In addition, several studies have shown that alterations in the RDW correlate with liver function and prognosis in numerous hepatic cancer-predisposing conditions, such as hepatitis B, chronic liver disease, and fibrosis [43,44,45,46,47]. Nevertheless, limited information is available regarding the prognostic role of the RDW in HCC, as well as its relationship with other blood-based biomarkers and the clinical characteristics of the disease. In this study, several clinical and blood-based biomarkers were evaluated, with the aim to investigate their ability to predict survival in patients with HCC, with a particular focus on RDW.

## 2. Materials and Methods

A consecutive series of patients with a histologic diagnosis of HCC attending the Department of Medicine, Surgery and Pharmacy of the University of Sassari, Italy, between 2009 and 2017, were included in this study irrespective of their age, stage of the disease, and treatment. Patients were followed-up for three years, without any censoring. Demographic and anthropometrical data [age, sex, body mass index (BMI)], as well as data on comorbidities and clinical status (Charlson Comorbidity Index, Child–Pugh score, etc.), along with laboratory tests were retrieved from patients’ clinical files and saved in a digital database. In addition, data regarding single (medical, surgical, or ablative) or combined treatments were collected. Pediatric patients, as well as those with no complete demographic, pathological, and clinical data were excluded. All patients were informed about the aims and the procedures of this study and provided written consent. This study was performed in accordance with the principles of the declaration of Helsinki and was approved by the ethical committee of the University Hospital of Cagliari (A.O.U Cagliari, Protocol number PG/2019/4493).

Results were reported as means and standard deviations (SDs) or medians and interquartile ranges (IQRs). Individual variables’ distribution was classified using the Shapiro–Wilk test. Statistical differences were investigated with the unpaired Student’s t-test or the Mann–Whitney rank-sum test, according to variable types. Univariate and multivariate logistic regression analysis was performed to assess correlations between individual variables and three-year mortality (primary end-point). Receiver operating characteristics (ROC) curve tests were carried out to identify the best cut-off values, maximizing sensitivity and specificity. Sensitivity and specificity were calculated using the optimal ROC curve value according to the Youden Index. For survival analysis, time zero was set as the time of diagnosis. Survival probability was investigated using the Kaplan–Meyer approach and the log-rank test, with death being the end point. Cox proportional hazards regression was carried out for both univariate and multivariate analyses. Hazard ratios were calculated from Cox analysis. Multivariate analyses were adjusted for those confounders that showed associations with *p*-values of <0.1 in univariate analysis. Statistical analyses were performed using MedCalc for Windows, version 20.014 64 bit (MedCalc Software, Ostend, Belgium).

## 3. Results

The characteristics of the 104 patients with HCC enrolled in this study are summarized in Table 1. At the end of the three-year follow-up, 54 of them (69%) passed away. Median age at diagnosis was 70 years (IQR 63–78 years). No statistically significant difference (*p* = 0.65) was found between the mean age of survivors (70 years, 64–75 years) and non-survivors (69 years, 62–79 years). There were 85 males (81.7%); 46 of them were (86.8%) in the survivor group and 39 (76.5%) in the non-survivor group (*p* = 0.18). There were no significant between-group differences in the BMI (*p* = 0.96) and the Charlson Comorbidity Index (*p* = 0.66). By contrast, there were significant differences between survivors and non-survivors in the Child–Pugh score (*p* = 0.02) and number of lesions (*p* = 0.008).

In addition, non-survivors had significantly higher values of the RDW (median: 15.1%; IQR: 14.2–16.6% vs. 13.7% IQR: 13.1–14.8%, *p* = 0.0001), total bilirubin (median: 1.20 mg/dL; IQR: 0.71–1.85 mg/dL vs. 0.90 mg/dL; IQR: 0.70–1.30 mg/dL, *p* = 0.04), and alkaline phosphatase (ALP) (median: 111 mg/dL; IQR: 82–156 mg/dL vs. 0.90 mg/dL; IQR: 0.70–1.30 mg/dL, *p* = 0.04). RDW was significantly associated with lower albumin (*p* = 0037) and higher total bilirubin concentrations (*p* = 0.0082); no correlations were observed between the RDW and BCLC stage (*p* = 0.4400), γ-GT (*p* = 0.5489), ALP (*p* = 0.0982), AST (*p* = 0.7479), or AST (*p* = 0.2676).

In univariate logistic regression (Table 2), there were significant associations between three-year mortality and Child–Pugh score (OR  =  4.5833; 95% CI 1.1970–17.5500, *p* = 0.0263), number of lesions (OR = 3.2621; 95% CI 1.604–4.322, *p* = 0.0001), treatment (OR = 0.4474; 95% CI 0.2185–0.9159, *p* = 0.0278), RDW (OR = 1.5727; 95% CI 1.2244–2.0200, *p* = 0.0004), total bilirubin (OR = 2.2813; 95% CI 1.1290–4.6097, *p* = 0.0216), and ALP (OR = 1.0099; 95% CI 1.0014–1.0185, *p* = 0.0228). In multivariate logistic regression (Table 3), the ORs for RDW (OR = 1.3877; 95% CI 1.0643–1.8093, *p* = 0.0155) remained significant after adjusting for the Child–Pugh score, lesion number, treatment, albumin, total bilirubin, and ALP.

**Figure 1:**
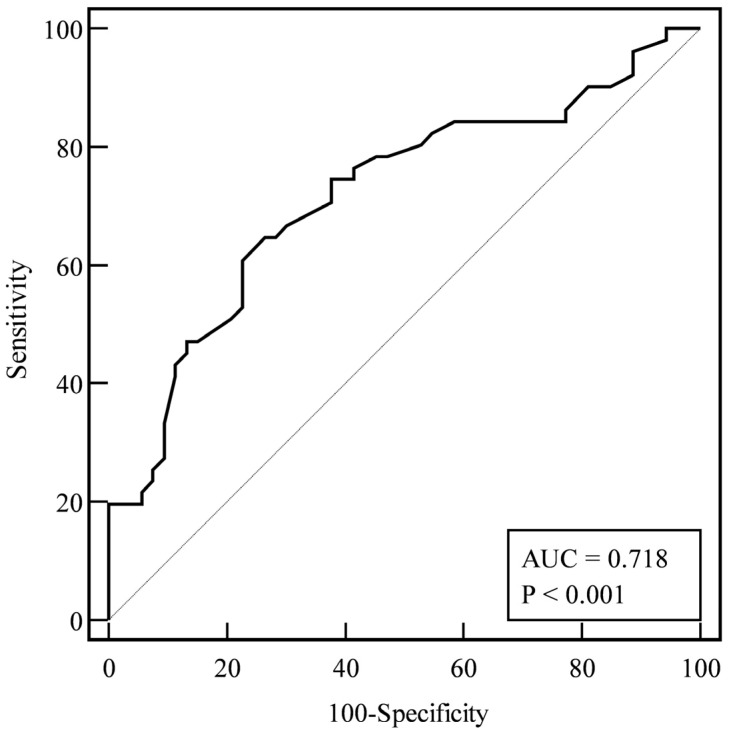
Receiver operating characteristics (ROC) curves for RDW as predictor of survival.

ROC curve analysis was performed to evaluate the sensitivity, specificity, and diagnostic accuracy of RDW in discriminating between survivors and non-survivors (Figure 1). With respect to survival, the optimal cut-off value identified with the Youden test was 14.7%, with 65% sensitivity and 74% specificity (AUC  =  0.718, 95% CI 0.622–0.802, *p*  <  0.001).

**Figure 2:**
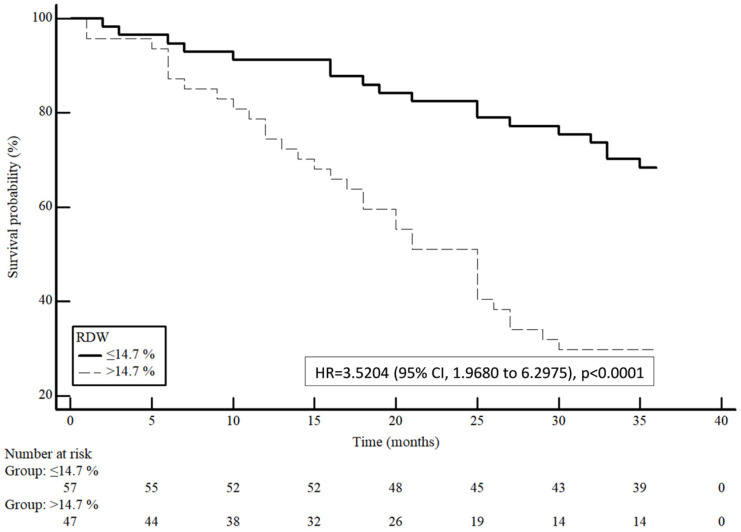
Kaplan–Meier curves for three-year survival probability of HCC in patients with different RDW levels.

The Kaplan–Meier survival curve, after classifying the patients based on Youden cut-off obtained via ROC curves (Figure 2), showed significantly lower survival with higher RDW values (HR = 3.5204; 95% CI 1.9680–6.2975, *p* < 0.0001), with a mean survival of 30.9 ± 9.67 months for patients with RDW ≤ 14.7% and 22.3 ± 11.4 months for patients with RDW > 14.7%. Three-year survival rate was 68% in the first group and 30% in the second.

In univariate Cox regression (Table 4), significant associations were found between survival and Child–Pugh score (HR  =  2.3517; 95% CI 1.1984–4.6146, *p* = 0.0129), treatment (HR = 0.5267; 95% CI 0.3049–0.9097, *p* = 0.0315), RDW (HR = 1.3932; 95% CI 1.2375–1.5686, *p* < 0.0001), albumin (HR = 0.6433; 95% CI 0.4150–0.9970, *p* = 0.0485), total bilirubin (HR = 1.2558; 95% CI 1.1101–1.4207, *p* = 0.0003), and ALP (HR = 1.0061; 95% CI 1.0015–1.0108, *p* = 0.0095). RDW (HR = 1.2524; 95% CI 1.0786–1.4542, *p* = 0.0032) and total bilirubin (HR = 1.2121; 95% CI 1.0155–1.4468, *p* = 0.031) remained significantly associated with survival after adjusting for the Child–Pugh score, Barcelona Clinic Liver Cancer (BCLC) stage, number of lesions, treatment, albumin, and ALP (Table 5).

## 4. Discussion

RDW is a simple and inexpensive parameter which reflects the degree of heterogeneity of erythrocyte volume (known as anisocytosis) and accelerated red cell turnover; it is traditionally used in laboratory hematology for the differential diagnosis of anemias [48]. Anisocytosis, defined as the presence of red blood cells (RBCs) with a broad heterogeneity of size and volume in peripheral blood, can be caused by numerous clinical conditions, including congenital erythrocyte disorders (i.e., β-thalassemia, sickle cell disease, hereditary spherocytosis), anemia (e.g., due to iron, folate, or vitamin B deficiencies), blood transfusions, some forms of hemolytic anemias, oxidative stress, inflammation, impaired renal function, and cancer [48]. RDW is calculated by dividing the standard deviation (SD) of erythrocyte volumes for the mean corpuscular volume (MCV) (i.e., RDW = SD/MCV). Although the result can be expressed either in absolute values (i.e., RDW-SD) or as a percentage (i.e., RDW-%), the latter is more widely used in routine laboratory practice and was the one adopted in this study [49].

RDW alterations in cancer may be due to various events, including the direct stimulation of red cell turnover by the tumors themselves, the immune responses to their dissemination, or to a state of chronic occult bleeding and anemia. Fancellu et al. recently described significant RDW differences between right-sided and left-sided colorectal cancers [31]. They hypothesized that the right colon, especially the caecum, is significantly larger than the left colon, and has less anatomical relations with neighboring organs in comparison to the rectum. This allows right-sided tumors to grow and remain clinically silent for a long time and explains why these lesions affect older patients and have an exophytic, ulcerated aspect with a higher stage at diagnosis. Older studies estimated that the time interval between cancer onset and clinical manifestations varies between 4.8 and 5.8 years [50]. During growth, the lesions become ulcerated and bleed. Chronic occult bleeding causes iron loss and thus chronic microcytic anemia. Bleeding is common in other long-lasting slowly growing solid tumors, especially those of the gastrointestinal, respiratory, and genitourinary tract, and this may be the reason for the differences in RDW values. In addition, central necrosis with intratumoral red cell consumption is common in solid parenchymal tumors, such as those of the liver and the intrahepatic biliary tract.

A few studies have investigated the role of the RDW in predicting prognosis in HCC. The first study was published in 2015 by Smirne et al. [51]. The authors retrospectively analyzed a training cohort of 208 patients with HCC and an independently prospectively collected validated cohort of 106 patients with HCC. In both cohorts, median survival time was significantly lower in patients with RDW ≥14.6% at the time of diagnosis. The median survival in the training cohort was 1026 days in the low RDW group (RDW ≤14.6%) vs. 282 days in the high RDW group (HR = 0.43; 95% CI: 0.31–0.60, *p* < 0.0001). The patients with HCC were then classified in four quartiles according to their RDW values, and the median survival decreased progressively with each increasing RDW quartile. In the validation cohort, the median survival was 868 days in patients with RDW <14.6%, whereas it was 340 days in patients with RDW >14.6%. In the training cohort, the survival rates at 1, 2, and 3 years were 79%, 57%, and 42% in patients with RDW <14.6% compared to 48%, 29%, and 18% in patients with RDW >14.6%, respectively. In both the training and validation cohorts, an increased RDW value was independently associated with higher mortality. Notably, the cut-off level identified by the authors (14.7%) is identical to the one identified in our study (14.7%).

Hu et al., in a study comprising 298 patients (including 81 HCC cases) and 66 healthy controls, showed that the RDW was associated with worse hospital outcomes (AUC 0.76 (95% CI 0.67–0.84) [43]. RDW values above 15.15% were independently associated with poor hospital outcomes after adjustment for serum bilirubin, platelet count, prothrombin time, albumin, and age, with an odds ratio of 13.29 (95% CI 1.67–105.68). In 2016, Zhou et al. investigated the relationship between the preoperative serum RDW value and the clinic and pathologic characteristics of 106 patients with HCC who underwent curative resection [30]. Two groups were identified based on a cut-off level that was very similar to the one described by Smirne et al., as well as in our study: high RDW (>14.5%, n = 28) and low RDW (≤14.5%, n = 78). The patients with preoperative high levels of RDW had significantly worse survival than those with low RDW (*p* < 0.05). According to multivariate analysis, high RDW (HR = 1.89, *p* = 0.002), TNM stage (HR = 1.70, *p* = 0.019), tumor size (HR = 1.33, *p* = 0.045), tumor number (HR = 1.42, *p* = 0.027), and vascular invasion (HR = 1.64, *p* = 0.009) were independent prognostic factors of overall survival in this study. The same year, Wei et al. retrospectively reviewed the medical records of 110 treatment-naive patients with HCC and 68 controls and found that the RDW was significantly increased in patients with HCC and correlated with the liver function tests [52]. No correlation between RDW and the stage of the disease was found in this study.

Subsequently, Howell et al. investigated the prognostic role of the RDW in patients with HCC treated with sorafenib [53]. A total number of 442 subjects were enrolled with a median follow-up time of 7.1 months. The baseline RDW was found to be significantly associated with survival (HR 1.23, 95% CI: 1.12–1.29). Interestingly, the authors found that the AUC markedly increased from 0.669 to 0.787 after the RDW, and no treatment-related diarrhea was considered as a factor when determining the CLIP score (Cancer of Liver Italian Program Score) for predicting 12-month survival. In a retrospective study encompassing 442 patients with HCC, Jing et al. reported that the RDW in patients with HCC with a tumor size larger than 10 cm was higher than that of patients with HCC with a tumor size smaller than 3 cm, 3–5 cm, and 5–10 cm (14.77 ± 2.35%, 15.27 ± 2.65%, 15.32 ± 2.40% vs. 15.97 ± 2.39%, *p* < 0.001); furthermore, the RDW level significantly increased with worsening Child–Pugh grades and BCLC stages [54]. Finally, they found that patients with HCC with RDW values below 14.15% (HR 0.530, 95 CI 0.395–0.710; *p* < 0.001) had lower mortality rates.

Golriz et al. investigated the role of preoperative, six- and twelve-month RDW values in predicting recurrence after curative resection for HCC in 395 patients [55]. The authors reported that recurrence-free survival (RFS) was significantly higher among patients with low RDW at 6 and 12 months, postoperatively (*p* < 0.001 and *p* = 0.028). In addition, RDW values higher than 16.15% at 6 months (HR: 2.047, *p* < 0.001) and higher than 15.85% at 12 (HR: 3.105, *p* < 0.002) months after liver resection were independent predictors of relapse-free survival. More recently, Tan et al. conducted a retrospective study comprising 745 patients with hepatitis B(HBV)-related HCC, 253 patients with chronic hepatitis B (CHB), as well as 256 healthy individuals, which included as a control group to enable a comparison to be made regarding hematological parameters and RDW values [56]. Potential risk factors for long-term all-cause mortality in patients with HBV-related HCC were predicted using multivariate Cox regression. The RDW of patients with HBV-related HCC was significantly higher when compared to CHB and healthy controls. In the former, splenomegaly, liver cirrhosis, larger tumor diameter, multiple tumor number, portal vein tumor thrombus, and lymphatic or distant metastasis were significantly increased, and a greater Child–Pugh grade and BCLC stage was correlated with a higher RDW. Furthermore, multivariate Cox regression analysis identified RDW as an independent risk factor for predicting long-term all-cause mortality in patients with HBV-related HCC. Finally, the authors generated a nomogram incorporating RDW with other clinical parameters and tested it in a training and a validation cohort; their nomogram showed high accuracy in predicting the survival and prognosis of patients with HBV-related HCC, suggesting its potential utility in clinical practice.

Despite a consistent methodological and treatment heterogeneity among these studies, they all showed that RDW has a potential predictive role in HCC prognosis. In our study, we included patients treated with different approaches. In addition, the cut-off level for a good predictive performance (AUC 0.718) found in our study was very similar to those described in other studies. Notably, the RDW maintained its predictive ability, even after we adjusted for numerous clinical factors, and performed better than other blood-based biomarkers. Although the mechanisms underpinning the superior performance of the RDW remain elusive, it has been hypothesized that a state of chronic inflammation and oxidative stress, typically associated with HCC, could explain the accelerated red blood cell turnover and anisocytosis [57].

Our study has some limitations, particularly regarding its retrospective design and the relatively small number of patients, which did not allow for consistent subgroup analyses to be performed in accordance to the treatment received by the patients. In addition, the data regarding the dimensions of the tumors and details on the underlying etiological causes were not analyzed. It must also be underlined that most of the patients included in this study had a low BCLC stage and Child–Pugh score, which may have negatively impacted the predictive capacity of the RDW. Further studies should include a proportion of patients with advanced-stage HCCs. On the other hand, our study shows the potential of RDW to independently predict prognosis in patients with HCC, regardless of the underlying etiology and treatment, and the ability to perform better than other biomarkers.

## 5. Conclusions

Our results show the potential of RDW to independently predict prognosis in patients with HCC, regardless of the treatment, and the ability to perform better than other biomarkers. Considering that RDW is a low-cost, easy-to-perform interpretable biomarker, its potential clinical application in this context is attractive. Nevertheless, such an application needs to be further evaluated in future prospective, randomized trials.

## Figures and Tables

**Table 1:** Demographic, clinical, and laboratory data of the whole population and at three years—survivor and non-survivor sub-groups.

	Total (*n* = 104)	Survivors (*n* = 53)	Non-Survivors (*n* = 51)	*p*-Value
Age (years)	70 (63–78)	70 (64–75)	69 (62–79)	0.65
Gender (F/M)	19/85	7/46	12/39	0.18
BMI	25.0 (23.3–27.6)	25.1 (23.4–27.5)	24.7 (23.1–27.8)	0.96
Charlson Comorbidity Index	7.6 ± 1.7	7.6 ± 1.8	7.7 ± 1.6	0.66
Child–Pugh score (A/B/C)	90/14/0	50/3/0	40/11/0	0.02
BCLC stage (0/A/B/C)	9/47/42/3	5/28/19/1	4/19/23/2	0.51
Lesion number (single/multiple)	72/32	43/10	29/22	0.008
Lesion size (<5 cm/≥5 cm)	72/32	37/16	35/16	0.90
Extrahepatic metastasis (no/yes)	102/2	52/1	50/1	0.98
Viral hepatitis	20/83	9/43	11/40	0.59
Treatment (medical/ablation/surgical/multiple)	5/66/31/2	0/32/20/1	5/34/11/1	0.054
MCV (fL)	87.7 ± 10.7	89.1 ± 8.9	86.3 ± 12.3	0.19
RDW (%)	14.5 (13.3–16.0	13.7 (13.1–14.8)	15.1 (14.2–16.6)	0.0001
PLT (×10^9^/L)	129 (88–194)	129 (93–189)	130 (86–201)	0.62
MPV (fL)	9.0 (8.1–10.1)	9.0 (8.1–10.4)	9.0 (8.0–9.9)	0.44
WBC (×10^9^/L)	5.5 (4.0–7.7)	5.2 (4.1–6.7)	5.9 (3.9–8.1)	0.47
Neutrophils (×10^9^/L)	3.1 (2.2–4.5)	3.0 (2.2–4.1)	3.1 (2.4–4.9)	0.44
Lymphocytes (×10^9^/L)	1.40 (1.00–2.10)	1.40 (1.10–2.02)	1.40 (0.82–2.08)	0.44
Monocytes (×10^9^/L)	0.40 (0.30–0.60)	0.40 (0.30–0.50)	0.40 (0.30–0.65)	0.44
CRP (mg/dL)	0.68 (0.22–3.53)	0.42 (0.19–3.98)	1.25 (0.44–2.87)	0.07
ESR (mm/h)	28.5 (13.4–48.0)	28.5 (11.0–40.0)	26.5 (13.0–53.0)	0.65
Albumin (g/dL)	3.5 (3.1–3.8)	3.5 (3.1–4.0)	3.4 (3.0–3.8)	0.15
Total bilirubin (mg/dL)	1.03 (0.70–1.50)	0.90 (0.70–1.30)	1.20 (0.71–1.85)	0.04
ALT (U/L)	48 (28–79)	50 (30–92)	47 (28–70)	0.37
AST (U/L)	54 (32–87)	50 (31–90)	54 (33–82)	0.95
γ-GT (U/L)	85 (52–137)	81 (48–133)	88 (53–154)	0.37
ALP (U/L)	102 (73–145)	87 (69–127)	111 (82–156)	0.02
LDH (U/L)	209 (175–263)	205 (178–276)	215 (172–240)	0.64
INR	1.12 (1.06–1.23)	1.11 (1.07–1.19)	1.16 (1.05–1.27)	0.24
Fibrinogen (mg/dL)	275 ± 83	277 ± 80	273 ± 87	0.84
Creatinine (mg/dL)	0.83 (0.74–1.01)	0.83 (0.77–1.02)	0.82 (0.72–1.01)	0.51
Urea (mg/dL)	34 (26–43)	35 (26–43)	32 (26–43)	0.92
AFP (ng/mL)	10.7 (4.7–47.8)	9.7 (4.7–23.7)	13.5 (4.7–86.1)	0.31

AFP: alpha fetoprotein; ALP: alkaline phosphatase; ALT: alanine aminotransferase; AST: aspartate aminotransferase; BCLC: Barcelona clinic liver cancer; BMI: body mass index; CRP: C reactive protein; ESR: erythrocyte sedimentation rate; F: females; INR: international normalized ratio; LDH: lactate dehydrogenase; M: males; MCV: mean corpuscular volume; MPV: mean platelet volume; PLT: platelets; RDW: red cell distribution width; WBC: white blood cell; γ-GT: gamma glutamyl transferase.

**Table 2:** Univariate logistic regression analysis assessing association between demographic, clinical, and laboratory data with three-year mortality.

	Odds Ratio	95% CI	*p*-Value
Age	1.0114	0.9736 to 1.0507	0.5595
Gender	2.0220	0.7254 to 5.6363	0.1783
BMI	1.0076	0.8611 to 1.1790	0.9248
Charlson Comorbidity Index	1.0363	0.8252 to 1.3014	0.7589
Child–Pugh score	4.5833	1.1970 to 17.5500	0.0263
BCLC stage	1.4614	0.8182 to 2.6102	0.1998
Lesion number	3.2621	1.3483 to 7.8922	0.0087
Lesion size	1.0571	0.4596 to 2.4315	0.8960
Extrahepatic metastasis	1.0400	0.0633 to 17.0838	0.9781
Viral hepatitis	0.7611	0.2855 to 2.0290	0.5853
Treatment	0.4474	0.2185 to 0.9159	0.0278
MCV	0.9757	0.9403 to 1.0124	0.1912
RDW	1.5727	1.2244 to 2.0200	0.0004
PLT	0.9995	0.9946 to 1.0045	0.8499
MPV	0.8775	0.6768 to 1.1378	0.3241
WBC	1.0372	0.8986 to 1.1972	0.6175
Neutrophils	1.0126	0.8600 to 1.1921	0.8809
Lymphocytes	0.8716	0.5620 to 1.3517	0.5392
Monocytes	2.8277	0.5483 to 14.5839	0.2143
CRP	0.9683	0.8474 to 1.1063	0.6353
ESR	1.0050	0.9866 to 1.0238	0.5975
Albumin	0.5296	0.2777 to 1.0100	0.0536
Total bilirubin	2.2813	1.1290 to 4.6097	0.0216
ALT	0.9973	0.9918 to 1.0029	0.3416
AST	1.0001	0.9937 to 1.0065	0.9837
γ-GT	1.0015	0.9980 to 1.0049	0.4079
ALP	1.0099	1.0014 to 1.0185	0.0228
LDH	0.9978	0.9933 to 1.0023	0.3311
INR	6.0213	0.4508 to 80.4309	0.1746
Fibrinogen	0.9995	0.9944 to 1.0046	0.8423
Creatinine	0.6328	0.1556 to 2.5732	0.5225
Urea	1.0108	0.9834 to 1.0389	0.4436
AFP	1.0001	0.9999 to 1.0003	0.2938

AFP: alpha fetoprotein; ALP: alkaline phosphatase; ALT: alanine aminotransferase; AST: aspartate aminotransferase; BCLC: Barcelona clinic liver cancer; BMI: body mass index; CRP: C reactive protein; ESR: erythrocyte sedimentation rate; F: females; INR: international normalized ratio; LDH: lactate dehydrogenase; M: males; MCV: mean corpuscular volume; MPV: mean platelet volume; PLT: platelets; RDW: red cell distribution width; WBC: white blood cell; γ-GT: gamma glutamyl transferase.

**Table 3:** Multivariate logistic regression analysis assessing association between studied variables and three-year mortality.

	Odds Ratio	95% CI	*p*-Value
Child–Pugh score	2.0049	0.2791 to 14.4038	0.4893
Lesion number	2.6633	0.9644 to 7.3551	0.0587
Treatment	0.5366	0.2265 to 1.2712	0.1572
RDW	1.3877	1.0643 to 1.8093	0.0155
Albumin	1.0818	0.4146 to 2.8227	0.8723
Total bilirubin	1.3584	0.7867 to 2.3455	0.2717
ALP	1.0068	0.9978 to 1.0159	0.0555

ALP: alkaline phosphate; RDW: red cell distribution width.

**Table 4:** Univariate COX regression analysis assessing association between studied variables and survival.

	Hazard Ratio	95% CI	*p*-Value
Age	1.0081	0.9805 to 1.0365	0.5679
Gender	1.5989	0.8365 to 3.0559	0.1556
BMI	1.0001	0.8823 to 1.1336	0.9990
Charlson Comorbidity Index	1.0288	0.8757 to 1.2087	0.7299
Child–Pugh score	2.3517	1.1984 to 4.6146	0.0129
BCLC stage	1.4584	0.9417 to 2.2587	0.0909
Lesion number	2.1653	1.2406 to 3.7789	0.065
Lesion size	1.0462	0.5789 to 1.8909	0.8810
Extrahepatic metastasis	1.0441	0.1438 to 7.5834	0.9660
Viral hepatitis	0.7452	0.3822 to 1.4529	0.3879
Treatment	0.5267	0.3049 to 0.9097	0.0215
MCV	0.9798	0.9535 to 1.0068	0.1413
RDW	1.3932	1.2375 to 1.5686	<0.0001
PLT	0.9997	0.9958 to 1.0036	0.8826
MPV	0.9070	0.7468 to 1.1016	0.3251
WBC	1.0232	0.9246 to 1.1324	0.6574
Neutrophils	1.0132	0.9055 to 1.1337	0.8192
Lymphocytes	0.8509	0.5986 to 1.2095	0.3681
Monocytes	1.7069	0.5764 to 5.0550	0.3344
CRP	0.9847	0.8967 to 1.0813	0.7468
ESR	1.0043	0.9911 to 1.0175	0.5269
Albumin	0.6433	0.4150 to 0.9970	0.0485
Total bilirubin	1.2558	1.1101 to 1.4207	0.0003
ALT	0.9978	0.9933 to 1.0022	0.3275
AST	0.9993	0.9947 to 1.0039	0.7559
γ-GT	1.0016	0.9994 to 1.0038	0.1619
ALP	1.0061	1.0015 to 1.0108	0.0095
LDH	0.9978	0.9941 to 1.0016	0.2635
INR	3.6363	0.6510 to 20.3116	0.1413
Fibrinogen	0.9992	0.9953 to 1.0032	0.7014
Creatinine	0.7547	0.2623 to 2.1714	0.6017
Urea	1.0086	0.9902 to 1.0272	0.3632
AFP	1.0000	1.0000 to 1.0001	0.3559

AFP: alpha fetoprotein; ALP: alkaline phosphatase; ALT: alanine aminotransferase; AST: aspartate aminotransferase; BCLC: Barcelona clinic liver cancer; BMI: body mass index; CRP: C reactive protein; ESR: erythrocyte sedimentation rate; F: females; INR: international normalized ratio; LDH: lactate dehydrogenase; M: males; MCV: mean corpuscular volume; MPV: mean platelet volume; PLT: platelets; RDW: red cell distribution width; WBC: white blood cell; γ-GT: gamma glutamyl transferase.

**Table 5:** Multivariate COX regression analysis assessing association between studied variables and survival.

	Hazard Ratio	95% CI	*p*-Value
Child–Pugh score	0.7474	0.2310 to 2.4178	0.6269
BCLC stage	1.2826	0.7455 to 2.2066	0.3687
Lesion number	1.4834	0.7648 to 2.8773	0.2433
Treatment	0.6985	0.3824 to 1.2758	0.2430
RDW	1.2524	1.0786 to 1.4542	0.0032
Albumin	0.7691	0.3999 to 1.4794	0.4316
Total bilirubin	1.2121	1.0155 to 1.4468	0.0331
ALP	1.0032	0.9975 to 1.0090	0.2762

ALP: alkaline phosphate; BCLC: Barcellona clinic liver cancer; RDW: red cell distribution width.

## Data Availability

Data are available from the corresponding author upon reasonable request.
